# Particle Swarm Optimization and Two-Way Fixed-Effects Analysis of Variance for Efficient Brain Tumor Segmentation

**DOI:** 10.3390/cancers14184399

**Published:** 2022-09-10

**Authors:** Naoual Atia, Amir Benzaoui, Sébastien Jacques, Madina Hamiane, Kaouther El Kourd, Ayache Bouakaz, Abdeldjalil Ouahabi

**Affiliations:** 1 Department of Electrical Engineering, University Mohamed Khider of Biskra, Biskra 07000, Algeria; 2 Electrical Engineering Department, University of Skikda, BP 26, El Hadaiek, Skikda 21000, Algeria; 3 University of Tours, 60 rue du Plat D’Etain, CEDEX 1, 37020 Tours, France; 4 College of Engineering, Royal University for Women, West Riffa 37400, Bahrain; 5 Department of Physics, Benyoucef Benkhedda University of Algiers, Algiers 16000, Algeria; 6 UMR 1253, iBrain, INSERM, Université of Tours, 37000 Tours, France

**Keywords:** brain tumor, image segmentation, PSO, ANOVA, K-means

## Abstract

**Simple Summary:**

Segmentation of brain tumor images from magnetic resonance imaging (MRI) is a challenging topic in medical image analysis. The brain tumor can take many shapes, and MRI images vary considerably in intensity, making lesion detection difficult for radiologists. This paper proposes a three-step approach to solving this problem: (1) pre-processing, based on morphological operations, is applied to remove the skull bone from the image; (2) the particle swarm optimization (PSO) algorithm, with a two-way fixed-effects analysis of variance (ANOVA)-based fitness function, is used to find the optimal block containing the brain lesion; (3) the K-means clustering algorithm is adopted, to classify the detected block as tumor or non-tumor. An extensive experimental analysis, including visual and statistical evaluations, was conducted, using two MRI databases: a private database provided by the Kouba imaging center—Algiers (KICA)—and the multimodal brain tumor segmentation challenge (BraTS) 2015 database. The results show that the proposed methodology achieved impressive performance, compared to several competing approaches.

**Abstract:**

Segmentation of brain tumor images, to refine the detection and understanding of abnormal masses in the brain, is an important research topic in medical imaging. This paper proposes a new segmentation method, consisting of three main steps, to detect brain lesions using magnetic resonance imaging (MRI). In the first step, the parts of the image delineating the skull bone are removed, to exclude insignificant data. In the second step, which is the main contribution of this study, the particle swarm optimization (PSO) technique is applied, to detect the block that contains the brain lesions. The fitness function, used to determine the best block among all candidate blocks, is based on a two-way fixed-effects analysis of variance (ANOVA). In the last step of the algorithm, the K-means segmentation method is used in the lesion block, to classify it as a tumor or not. A thorough evaluation of the proposed algorithm was performed, using: (1) a private MRI database provided by the Kouba imaging center—Algiers (KICA); (2) the multimodal brain tumor segmentation challenge (BraTS) 2015 database. Estimates of the selected fitness function were first compared to those based on the sum-of-absolute-differences (SAD) dissimilarity criterion, to demonstrate the efficiency and robustness of the ANOVA. The performance of the optimized brain tumor segmentation algorithm was then compared to the results of several state-of-the-art techniques. The results obtained, by using the Dice coefficient, Jaccard distance, correlation coefficient, and root mean square error (RMSE) measurements, demonstrated the superiority of the proposed optimized segmentation algorithm over equivalent techniques.

## 1. Introduction

### 1.1. What Is a Brain Tumor?

A brain tumor is a cluster of uncontrolled cancer cells that grow in or around the brain. Brain tumors are divided into two categories: primary tumors, originating in the brain or spinal cord, and secondary tumors, also called brain metastases, which develop elsewhere in the body and spread to the brain [1]. In the first category (i.e., primary tumors), the probability of a person developing this type of tumor in their lifetime is less than 1% [2]. This probability is low; however in 2020, for example, it still represented just over 308,000 people diagnosed worldwide [3]. A figure that should also alert us, is the increased incidence of brain tumors at all ages over the last 20 years. For example, the incidence has increased by more than 40% in adults. In the second category (i.e., secondary tumors), the cancers that most often spread to the brain are breast, kidney, and lung cancers, as well as leukemia, lymphoma, and melanoma [4].

A brain tumor can take many forms; it is therefore difficult for radiologists and physicians to diagnose it indisputably, because medical imaging images can vary in intensity. Several approaches for detecting and segmenting brain tumors from magnetic resonance imaging (MRI) images have been proposed in the literature, to help practitioners make their diagnoses [5,6].

In addition to MRI, functional ultrasound is a modality that is gaining recognition in medicine. Functional ultrasound can allow the imaging of the neuronal activity of the brain in small, awake, and mobile animals. Nevertheless, such a modality requires long ultrasound acquisitions at high frequency, to have an acceptable sensitivity; hence, possible material constraints [7].

During brain tumor surgery, two types of difficulties may arise: (i) identification of the tumor and its boundaries related to the healthy brain; (ii) identification of functional brain regions, i.e., those involved in neurological functions (skills, sensitivity, language, vision, cognition, etc.). The standard gold method currently used to improve the quality of brain tumor resection, while minimizing neurological risk, is so-called ‘standby’ surgery with direct electrical brain stimulation. Practitioners commonly use ultrasound to localize the tumor in the brain; however, to date, there are no pre- or intra-operative imaging tools to identify functional brain regions [8]; hence the need for innovative imaging in this area, such as high-frequency Doppler ultrasound, in the surgical management of patients with awake brain tumors. Ultrahigh frequency achieves a spatial resolution of 30 μm, and is thus more than five times better than MRI. The Doppler mode [9,10,11,12] detects microvascular flows at velocities less than 1 mm/second.

Gliomas are the most common primary brain tumors in adults. Nearly 3000 new cases are diagnosed each year in France. Men are more frequently affected. Most cases are sporadic, but in rare cases they are associated with certain family cancers [13,14].

About 75% of gliomas diagnosed are high-grade (III or IV of the World Health Organization (WHO) classification) [15].

Gliomas can develop in any region of the brain. They progressively infiltrate the brain parenchyma, and cause a mass effect.

Today, if the clinical examination should suggest a tumoral process, the diagnosis of a brain tumor relies on magnetic resonance imaging (MRI), due to its shooting in all orientations, its intrinsic 3D, its non-use of ionizing radiation, and its precision.

### 1.2. MRI Sequences for Brain Tumors

Brain MRI, with or without the injection of contrast products such as gadolinium, is systematic in cases of a suspected brain tumor. Brain MRI enables:

- the localization of the expansive process of the tumor, and the specification of its local extension;
- the specification of its characteristics, e.g., is it homogeneous or heterogeneous; is there perilesional edema, calcifications, necrosis, or intratumoral hemorrhage?;
- the establishment of a differential diagnosis between a brain tumor and a circumscribed lesion of another nature, e.g., an abscess;
- the establishment of the diagnosis of certain evolving tumor complications (hemorrhage, hydrocephalus, tumor meningitis, etc.).
- the establishment of the histological grade, in cases of a glial tumor;
- the definition of the quality of the tumor removal, and the continuation of the therapeutic strategy after the surgical time.

The most common MRI sequences are T1- and T2-weighted scans, where T1 and T2 are tissue-specific time constants.

T1-weighted images are produced using short TE and TR times, and vice versa for T2-weighted images, where TR is the repetition time, defined as the time interval between two excitations, and TE is the echo time, defined as the interval between the excitation and the appearance of the MRI signal. Generally, T1- and T2-weighted images can be easily differentiated, by observing the cerebrospinal fluid (CSF). The CSF is dark on T1-weighted images, and clear on T2-weighted images.

A third sequence that we will use in our work is the FLAIR sequence (i.e., fluid attenuated inversion recovery), which is an inversion-recovery sequence well-adapted to brain imaging, in which the cerebrospinal fluid signal is suppressed or strongly attenuated, and a long TE is used, to give it a solid T2 weighting. The FLAIR sequence has significantly improved the detection of brain parenchymal lesions, particularly those located at the parenchymal–CSF interface. White-matter pathologies (softening, demyelination processes, etc.) appear hyperintense. This sequence is particularly interesting for the early diagnosis of ischemic events; it allows us to obtain an image of excellent definition in a few minutes and can, contrary to the diffusion or perfusion sequences that we will not use in this work, be performed on all MRI machines. Currently available in 3D volume acquisition, it is part of the basic MRI workup of the brain.

Table 1 compares T1, T2, and FLAIR sequences in the context of brain tissue consisting of gray matter, CSF, and white matter.

**Figure 1:**
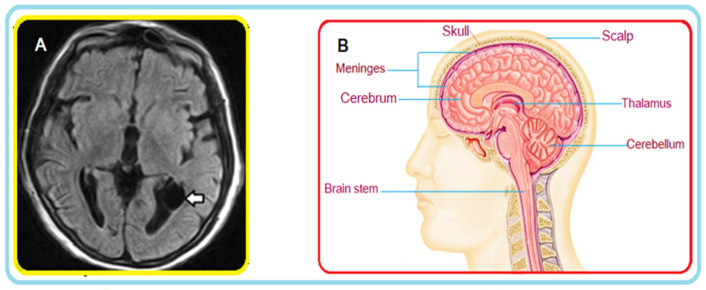
(**A**) MRI of the brain: axial FLAIR section. A thin-walled cyst-like image (arrow) consistent with an ependymal cyst can be seen in the occipital extension of the left lateral ventricle. (**B**) Brain cross-section (illustration).

Figure 1A shows an example of a FLAIR MRI sequence where a cyst is highlighted (see arrow); Figure 1B illustrates a cross-section in the axial plane of the human brain. Interestingly, the FLAIR sequence is very ‘sensitive’ to pathology, and clearly detects the cyst.

**Figure 2:**
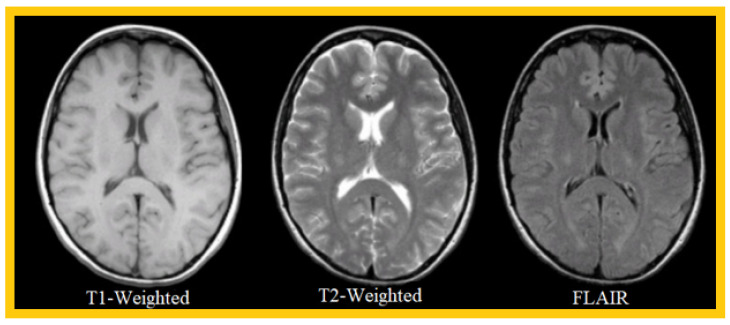
Example of 3 MRI sequences: T1-Weighted, T2-Weighted, and FLAIR.

**Figure 3:**
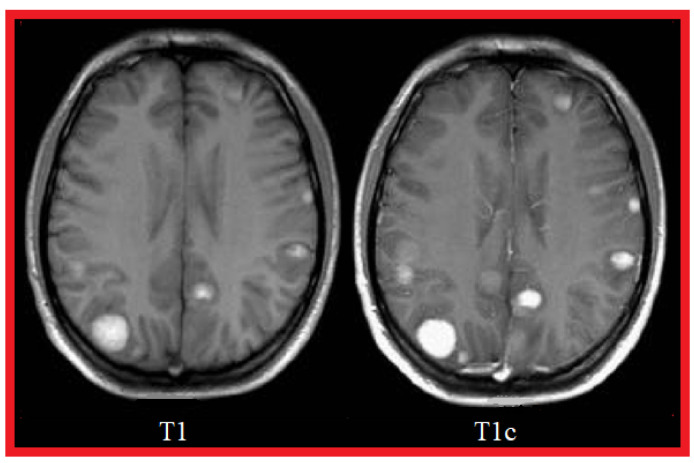
Comparison between a T1-Weighted MRI sequence without a contrast agent (T1) and the same sequence with a contrast agent (T1c).

Figure 2 shows an example of three MRI sequences: T1-Weighted, T2-Weighted, and FLAIR; in Figure 3, we have represented a T1-Weighted MRI sequence (noted simply ‘T1’) with and without contrast medium. The T1 sequence with contrast medium is noted ‘T1c’—sometimes noted ‘T1-Gd’, when the contrast agent is Gadolinium. This image represents a metastatic malignant melanoma. T1-weighted MRI shows multiple secondary lesions that are spontaneously hyper-signal. After injection of a contrast medium, the T1c image shows that the lesions have been enhanced, and are therefore better visualized, and new lesions are detected.

**Figure 4:**
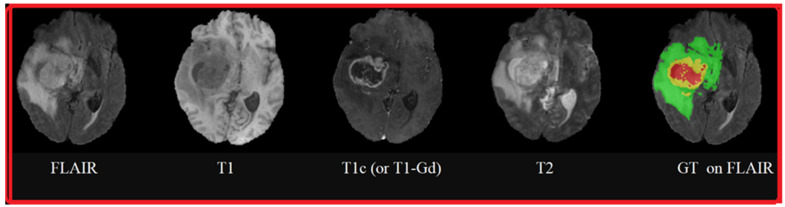
MRI sequences of brain tumors: FLAIR, T1, T1 contrasted by Gadolinium injection, T2, and Ground Truth superimposed on the FLAIR sequence.

Figure 4 shows an example of MRI sequences from the BraTS 2015 database [16], representing brain tumor pathologies. These sequences are FLAIR, T1, T1c (or, more precisely, T1-Gd, because the contrast agent used is Gadolinium), T2, and Ground Truth performed by specialists on which the FLAIR sequence is superimposed. The colors represent classes of tumors: red for necrosis; green for edema; and yellow for tumor.

### 1.3. Why We Should Be Interested in Brain Tumor Segmentation?

Image segmentation is the action of grouping pixels according to predefined criteria, in order to build regions or classes of pixels. There are several methods of image segmentation: methods based on contours, regions, classification, or hybrid. Segmentation and its automation remain today one of the major challenges in MRI, mainly in relation to brain tumor images, in order to help the practitioner in his daily practice, in the presence of a huge volume of images. These segmentation methods have long been manual or semi-automatic but, with the advent of new methods, some are now fully automated.

The segmentation of brain tumors is vital, because the patient’s life depends on it; the direct implication of our research is to propose efficient and safe methods to respond to this delicate operation fully. Indeed, the segmentation of brain tumors from MRI images, presented in our work, has direct practical implications for establishing an efficient diagnosis, following the tumor’s progression, or for evaluating the relevance of the prescribed treatment and therapy. It is well known that manual segmentation and analysis of structural MRI images of brain tumors is tedious and time-consuming; therefore, automated and robust segmentation of brain tumors will have a significant impact on disease management, by providing essential information about the nature, volume, location, and shape of the tumor [17,18].

**Figure 5:**
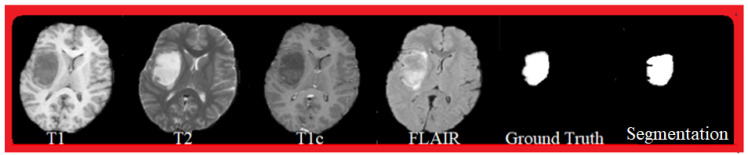
Segmentation result (see Section 4): MRI sequences: T1, T2, T1c, FLAIR, Ground Truth, and segmentation.

Figure 5 shows an example of successful segmentation. The Ground Truth segmentation is a manual segmentation performed by experts, and globally it corresponds to the proposed automatic segmentation (see Section 4 on the experimental analysis). The visualized MRI sequences are from the BraTS 2015 database, and correspond to T1, T2, T1c, and FLAIR sequences.

**Figure 6:**
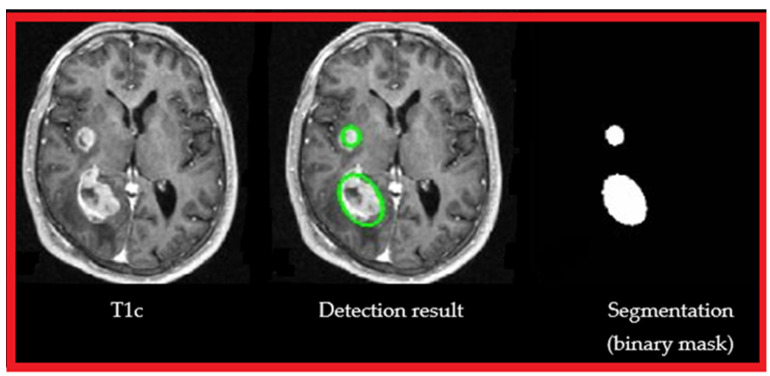
Segmentation result (see Section 4): T1c, automatic detection of brain tumors, segmentation, and binarization.

Figure 6 shows the detection result (center image) of our approach, from a T1 MRI sequence with contrast medium (left image) and segmentation (in green) binarization (right image).

### 1.4. Brain Tumor Segmentation Algorithms

As reported in [19], brain tumor segmentation algorithms can be grouped into three main categories: (1) conventional techniques; (2) classification and clustering techniques; and (3) deformable model techniques [20,21]. Threshold-based techniques, which compare pixel intensity to one or more intensity thresholds, belong to the first category: conventional techniques [22,23]. For example, an Otsu thresholding approach, combined with some morphological operations (i.e., dilation and erosion), was proposed in [24], to detect brain tumor diseases from MRI images. An extended method that would give more accurate thresholding performance was proposed in [25]. In addition, region-based techniques, in which disjoint regions are formed by merging neighboring pixels, based on a similarity criterion, are also classified in the first category [26]; these techniques include region-growth and watershed segmentation techniques. For example, an adaptive region-growing approach was proposed in [27], to solve the problem of manual threshold selection and weakness against noise; this approach was based on the variances and gradients of the inter- and intra-boundary curves. In another work, [28], the authors presented a region-growth approach based on a fixed threshold value for MRI segmentation, enhanced by an efficient pre-processing framework. In work presented by Biratu et al. [29], the authors modified the principle of the classical region-growing segmentation method; they applied it to the detection of abnormality regions in brain images. The seed point initialization, in the suggested method, was designed to be generated automatically for any input brain images, in contrast to the classical approach, where the seed point should be initialized manually. Khosravanian et al. [30] suggested a level set segmentation technique, based on the super-pixel fuzzy clustering and lattice Boltzmann method for autonomously segmenting brain tumors, which is strongly resistant to image intensity and noise.

The second category of brain tumor segmentation algorithms—classification and clustering techniques—includes several effective algorithms [31], such as K-means, support vector machines (SVMs), Markov random fields, artificial neural networks, convolutional neural networks (CNNs), and fuzzy C-means. For example, an SVM classification scheme, combined with a kernel-space feature selection methodology, was proposed in [32] for brain tumor segmentation. An unsupervised framework, based on random forests, was proposed in [33], to extract the tumor location, followed by a pattern classification phase. To define the tumor area, 86 features were used to create a training dataset, to be presented as input to the classifier. Currently, CNNs, or deep-learning-based models, have performed impressively in several medical imaging applications [34,35,36], as they assist in understanding complex patterns precisely. For example, a fully supervised system for brain tumor segmentation, based on a CNN architecture that exploits local and global contextual features, was proposed in [37]. In a recently published paper [38], fast convolutional neural networks were used to train, classify, and distinguish tumor from non-tumor patterns; training focused on patches and slices of axial, coronal, and sagittal brain views. Hussain et al. [39] created a correlation architecture of a parallel CNN layer and a linear CNN layer, by including an induction structure. Segmenting MR images of brain tumors, using this structure, has produced positive results. Using unpaired adversarial training, Li et al. [40] developed the innovative framework called TumorGAN, to provide efficient image segmentation of pairs. They added a regional perceptual loss, to improve the discriminator’s performance and the quality of the output images. Additionally, they created a localized L1 loss, to limit the color of the observed brain tissue. Arora et al. [41] proposed an automatic system to tackle the task of segmenting gliomas from MRI scans, based on a U-Net-based deep-learning model. Before presenting the input image to the deep model, the system transformed it by applying various approaches, including feature scaling, subset division, restricted object region, category brain slicing, and watershed segmentation. An approach for automatic segmentation, based on texture and contour, was proposed by Nabizadeh and Kubat [42]. The machine learning classifier was trained using landmark points, after determining high-level features. In the present work, we opted for K-means, which is an unsupervised learning model applying non-hierarchical data partitioning. This algorithm categorizes the data into multiple clusters, respecting the principle of exclusivity of membership: a single observation can only belong to one cluster. The advantage of K-means lies in its simplicity and the fact that it is used daily in the socio-economic world for data segmentation.

The third category of brain tumor segmentation algorithms consists of deformable model techniques, including parametric and geometric deformable models. These techniques have been proposed, among other things, to support intuitive interaction and high variability of mechanisms. For example, metaheuristic techniques have also been used for brain tumor segmentation, exploiting their ability to solve challenging optimization problems in minimal time, while avoiding local optima. Several techniques for brain tumor segmentation based on metaheuristics have been reported in the literature [43,44]. In [43], the Cuckoo search algorithm, an efficient optimization model, was applied to brain tumor segmentation, from MRI images. The ant colony optimization metaheuristic and a fuzzy classification approach were combined in [44], to segment and extract the suspicious region from the brain MRI image containing the tumor position. In a recent paper, [6], the authors proposed a novel region of interest (ROI)-based brain tumor segmentation method. The region of interest was first identified, using grid decomposition, and then only the region of interest was segmented, using the spectral clustering method. Segmenting the brain tumor from a region of interest, rather than from the entire image, is an attractive concept. Nevertheless, this approach was limited by the increased computational complexity resulting from the ROI identification step. In this study, we performed an analysis of variance (ANOVA) on the data, to quantify the differences between the results. ANOVA is a statistical method generally used to assess the similarity of means in different groups, by comparing variances [45,46]. The main advantages of ANOVA over other statistical methods lie in the following four points:

1. It is easy to implement, using simple algebra.
2. It can be used to compare more than two samples.
3. It can be applied to groups with different numbers of observations.
4. It has been widely used, and has proven effective in various research fields, such as pharmacology and medicine.

### 1.5. Main Contributions

This paper proposes an original brain tumor segmentation method, based on the particle swarm optimization (PSO) technique that uses fixed two-way ANOVA as the fitness function. The segmentation of a brain tumor is vital, because the patient’s life depends on it, and therefore the fundamental motivation of our work was to identify efficient and safe methods of responding to this delicate operation fully. This objective was achieved by our choice of PSO with ANOVA. The proposed algorithm consisted of three main steps:

1. The first step was to remove the skull bones from the image, to eliminate unnecessary information.
2. In the second step, which was the main contribution of this study, the PSO technique was applied, to detect the lesion’s brain image block. The two-way fixed ANOVA technique used a fitness function to determine the best among all candidate blocks, resulting in automatic brain tumor segmentation comparable to the Ground Truth performed by radiologists. All image blocks were tested, and the one that gave the minimum variance was considered. To overcome the computational complexity, PSO was used as a metaheuristic technique, that identified the best block in minimum time. The choice of PSO was based on the high performance of this optimization technique, when applied to many real-world applications. The satisfactory solution to a complex optimization problem, which includes many sub-optimal solutions, justified using a powerful metaheuristic, like PSO. The PSO algorithm, which is simple to understand, program, and use in minimal time, is particularly effective for practical optimization problems, such as image segmentation [47]. Therefore, the problem was posed as a maximization of a fitness function, and the well-known ANOVA method was chosen to measure the variance between the candidate block and the non-diseased block.
3. In the final step, K-means clustering—an efficient and straightforward partitioning technique—was applied to the lesion block, to classify it as tumor or non-tumor.

Our approach was original; to date, no other research has considered the techniques that were chosen, to provide a satisfactory answer to the segmentation of brain tumors. Therefore, the strengths of our approach were the originality and the quality of the results obtained.

To illustrate the essential role of ANOVA in the proposed algorithm, the experimental results obtained with the ANOVA-based fitness function were compared to those obtained with the well-known dissimilarity criterion, the sum of absolute differences (SAD). The comparison results—set against classical segmentation algorithms and recently published papers (i.e., state-of-the-art approaches), and using a private database provided by the Kouba imaging center, Algiers (KICA), and the multimodal brain tumor segmentation challenge (BraTS) 2015 database—demonstrated the efficiency and robustness of ANOVA.

The remainder of the paper is organized as follows: Section 2 presents the background of the method we propose here; in Section 3, the proposed algorithm is presented in detail, as well as the reasons for using PSO and ANOVA as the underlying techniques; the experimental results, and a comparison to the state-of-the-art, are presented in Section 4; a conclusion, summarizing the work, is given in Section 5.

## 2. Review of the Background of the Proposed Approach

The employed algorithm used different methods to segment the brain tumor image. The PSO metaheuristic technique was applied to identify the ROI, and the fitness function that determined the best block that could be considered as ROI was based on ANOVA. Finally, the K-means method was used to segment the ROI. This section presents the main concepts of these methods in detail.

### 2.1. Particle Swarm Optimization

Metaheuristic techniques have been developed to solve complex optimization problems, when mathematical techniques fail or require high computational time. The process of any metaheuristic technique starts with one or more random solutions initialized in the search-space. Then, powerful tools, inspired by natural phenomena, are used to iteratively converge the solution(s) to the optimal solution. The success of a metaheuristic technique relies on its ability to explore the search-space in depth, and to exploit promising areas.

Metaheuristics are grouped into three categories, based on the type of inspired natural phenomenon: (1) evolutionary algorithms inspired by genetic inheritance for survival, such as genetic algorithms (GAs) [48]; (2) swarm intelligence that mimics the social behavior of a group of animals, such as particle swarm optimization (PSO) [49]; (3) physics-based techniques, which are based on physical rules, such as simulated annealing (SA) [50]. Among the many metaheuristics developed in the literature, PSO has proven its efficiency in many applications [51,52].

PSO is a swarm intelligence technique proposed by Eberhart and Kennedy in 1995 [49], inspired by the flight behavior of birds. It has been successfully used in many application areas, due to its simplicity of implementation and effectiveness.

In PSO, many particles or candidate solutions are randomly initialized in the search-space, and then each particle iteratively adjusts its position, according to its own and its colleagues’ flight experience [49,53,54]. PSO’s fundamental concept is to randomly initialize $N_{p}$ candidate solutions in the search-space. Their velocities and positions are then updated, using (1) and (2):

(1) $$V_{i}=\omega \times V_{i}+c_{1}\times R_{1}\times \left(P_{i}-X_{i}\right)+c_{2}\times R_{2}\times \left(G-X_{i}\right)$$

(2) $$X_{i}=X_{i}+V_{i}$$

where $V_{i}$ and $X_{i}$ are the velocity and position of particle i, and $P_{i}$ and G are the individuals and global solutions; $R_{1}$ and $R_{2}$ are two random numbers, and ω, $c_{1}$, and $c_{2}$ are the weights of physical, cognitive, and social influences, respectively—they control the exploration and exploitation phases in PSO.

Each candidate solution is evaluated through a fitness function. The individual and global best candidate solutions are updated, based on the fitness function values.

A pseudo-code for PSO is described in Algorithm 1:

**Algorithm 1.** PSO algorithm
1: Initialize the total number of candidate solutions $N_{p}$, and the maximum number of iterations
$t_{max}$
2: Random initializaiton of candidate solutions 3: **for**
t=1:
$t_{max}$
**do** 4:   Update the velocities *V* with (1)
5:   Update the positions *X* with (2)
6:   Evaluate the positions *X* with the fitness function
7:   Update the individual $\left(P_{i}\right)$ and global (*G*) solutions
8: **End for**

### 2.2. Analysis of Variance (ANOVA)

Analysis of variance (ANOVA) is a statistical method developed by Larson in 2008 [45], to analyze the variation of a response variable determined under different conditions, defined by discrete factors. More precisely, the principle of ANOVA is to determine, using a statistical test, whether the share of dispersion attributable to the factor under study is significantly greater than the residual share. If the factorial dispersion is significantly larger than the residual dispersion, it means that the dispersion of the data around the means of each modality is small, compared to the dispersion of the means around the overall mean. In this case, if the means relative to each modality are highly dispersed, while the intra-class variability is low, it means that the means are globally different. Conversely, if the factorial dispersion is of the same order of magnitude as the residual dispersion, it means that the means are not different overall. Finally, the ANOVA is used to test the equality of the means of the different groups, by comparing their variances.

The ANOVA technique has two models that we will detail: the one-way fixed-effects ANOVA and the two-way fixed-effects ANOVA [46].

#### 2.2.1. One-Way Fixed-Effects ANOVA

The results of a one-way ANOVA are only significant if the following three assumptions are met:

- Each sample comes from a normally distributed population.
- The variances of the populations from which the samples come are equal.
- The observations in each group are independent, and the observations in the groups were obtained by random sampling.
  The null and alternative hypotheses defined in a one-way ANOVA are as follows:
- Null hypothesis or $H_{0}$: the means of the k groups in the study population are equal.
- Alternative hypothesis or $H_{1}$: at least one group means differs from the others.

A statistical test—for example, Fisher’s F test, with $\left(k-1\right)\;$ [factorial part] and $\left(N-k\right)$ [residual part, where N is the size of the study population] degrees of freedom (provided that the normality and homogeneity of the residuals are respected), at a given α risk (generally 5%)—allows us to reject, or not to reject, the null hypothesis. The key elements of the one-way ANOVA method are summarized in Table 2. The main definitions associated with this table are defined below:

- The total sum of squares (SST) is the sum of the squared distances between each observed value and the overall mean; it is the sum of weight attributable to the factor (SSF) and weight attributable to the residues (SSE); it can therefore be summarized by (3).
- The factorial sum of squares (SSF), which measures the differences between the group averages and the overall average, is defined in (4).
- The residual sum of squares (SSR) is defined in (5).
- p is the p-value corresponding to $F_{k-1,N-k}$.

Ultimately, let us suppose that the p-value is less than the threshold value that has been defined (usually 5%): in that case, the null hypothesis can be rejected, implying that at least one of the means in a population group differs from the others.

(3) $$SST=SSF+SSE=\overset{k}{\underset{i=1}{\sum }}\overset{n_{j}}{\underset{j=1}{\sum }}{\left(y_{ij}-\bar{y}\right)}^{2}$$

- SSF: factorial sum of squares.
- SSE: error sum of squares.
- i: index of the modalities (groups), i.e., from 1 to k.
- j: observation index in a modality.
- $y_{ij}$: observations.
- $\bar{y}$: overall mean of observations.

(4) $$SSR=\overset{k}{\underset{i=1}{\sum }}\overset{n_{j}}{\underset{j=1}{\sum }}{\left(y_{ij}-\bar{y_{i}}\right)}^{2}$$

(5) $$SSF=\overset{k}{\underset{i=1}{\sum }}n_{i}{\left(\bar{y_{i}}-\bar{y}\right)}^{2}$$

- $n_{i}$: numbers of data for each of the modalities.
- $\bar{y_{i}}$: mean of the $n_{i}$-values of the considered modality.

**Table 2:** Key elements of one-way ANOVA calculations.

Source of Variation	Sum of Squares	Degrees of Freedom	Mean Squares	F-Value	p-Value
Factor	SSF (attributable to factor)	k−1	$\frac{SSF}{k-1}$		
Residues or error	SSE (attributable to residues)	N−k	$\frac{SSE}{N-k}$	$F_{k-1,N-k}=\frac{\frac{SSF}{k-1}}{\frac{SSE}{N-k}}$	p
Total	SST=SSF+SSE	N−1			

#### 2.2.2. Two-Way Fixed-Effects ANOVA

The principle of the two-way fixed effects ANOVA model is, first, to decompose the total dispersion of the data into four sources: the contribution attributable to the first factor, noted A; the contribution attributable to the second factor, noted B; the contribution attributable to the interaction of the two factors; and the unexplained or residual share. In this method, each level of a factor is combined with the other factor: the two factors are said to be crossed. Then, in a second step, it is necessary to evaluate, with the help of a statistical test, if the factorial shares, and the one linked to the interaction, are significantly higher than the residual share.

Before going into the details of the method, it is necessary to define the following parameters:

- The first categorical variable studied (often called factor A) has a modalities (we also say that the factor A contains a levels). The index of the modalities of this first categorical variable, noted i, goes from 1 to a. Similarly, the second categorical variable studied (often called factor B) has b modalities. The index of the modalities of this second categorical variable, noted i, goes from 1 to b.
- The total number of observations is always noted as N, but the number of observations in each cell of the factorial design is noted as $n_{ij}$. Equation (6) defines the relationship between N and $n_{ij}$. In the following, n is the number of repetitions.
- The observations in each cell of the factorial design (i.e., in each factorial combination) are denoted by $y_{ijk}$, where k is the replication index in each crossover. The overall average of the responses is defined in (7), where the two points (“_.._”) correspond to the indices of the first and second categorical variables.
- The means of each cross of modalities are noted $\bar{y_{lJ}}$ (see (8)).
- The marginal means of the modalities of the first variable and those of the second variable are respectively noted $\bar{y_{l\cdot }}$ and $\bar{y_{\cdot J}}$, and meet the definitions of Equations (9) and (10).
- The marginal numbers of the modalities of the first variable and those of the second variable are respectively noted as $n_{i\cdot }$ and $n_{\cdot j}$, and meet the definitions of Equations (11) and (12).

(6) $$N=\overset{a}{\underset{i=1}{\sum }}\overset{b}{\underset{j=1}{\sum }}n_{ij}$$

(7) $$\bar{y_{..}}=\frac{1}{N}\overset{a}{\underset{i=1}{\sum }}\overset{b}{\underset{j=1}{\sum }}\overset{n_{ij}}{\underset{k=1}{\sum }}y_{ijk}$$

(8) $$\bar{y_{lJ}}=\frac{1}{n_{ij}}\overset{n_{ij}}{\underset{k=1}{\sum }}y_{ijk}$$

(9) $$\bar{y_{l\cdot }}=\frac{1}{b}\overset{b}{\underset{j=1}{\sum }}\bar{y_{lJ}}$$

(10) $$\bar{y_{\cdot J}}=\frac{1}{a}\overset{a}{\underset{i=1}{\sum }}\bar{y_{lJ}}$$

(11) $$n_{i\cdot }=\overset{b}{\underset{j=1}{\sum }}n_{ij}$$

(12) $$n_{\cdot j}=\overset{a}{\underset{i=1}{\sum }}n_{ij}$$

Like the one-way ANOVA method, and as shown in Table 3, the two-way ANOVA technique first measures the total dispersion of the data by calculating the total sum of squares (SST), as defined in (13). Then, the total dispersion is decomposed into the part attributable to the first factor, noted SSA (see (14)), the part attributable to the second factor, noted SSB (see (15)), the part attributable to the interaction between the two factors, noted SSAB (see (16)), and finally, the part attributable to the residues, noted SSR (see (17)). After calculating the variances of the factors, interaction, and residuals—which are referred to as mean squares in Table 3—statistical hypothesis tests, i.e., F-tests of the ratio of two variances, are performed, to assess whether each of the three variance shares is significantly greater than the residual variance. Under the assumptions of normality and homogeneity of residuals, the F-test statistic follows a Fisher distribution with:

- $\left(a-1\right)$ and $ab\left(n-1\right)$ degrees of freedom for the tests related to factor A;
- $\left(b-1\right)$ and $ab\left(n-1\right)$ degrees of freedom for the tests related to factor B;
- $\left(a-1\right)\left(b-1\right)$ and $ab\left(n-1\right)$ degrees of freedom for the tests related to the interaction between the two factors, A and B.

Finally, as in the previous section, and in a classical way, we must calculate the probability, under the null hypothesis $H_{0}$, of observing such an F-value: this is the p-value. This p-value is compared to the chosen level of significance (generally set at 5%). If the p-value is lower than the significance level, we conclude that the effect is significant. If not, the conclusion is not that there is no effect, but that there is no evidence of an effect. It is possible, for example, that the sample sizes are too small to show a significant difference.

(13) $$SST=\overset{a}{\underset{i=1}{\sum }}\overset{b}{\underset{j=1}{\sum }}\overset{n_{ij}}{\underset{k=1}{\sum }}{\left(y_{ijk}-\bar{y_{..}}\right)}^{2}$$

(14) $$SSA=bn\overset{a}{\underset{i=1}{\sum }}{\left(\bar{y_{l\cdot }}-\bar{y_{..}}\right)}^{2}$$

(15) $$SSB=an\overset{b}{\underset{j=1}{\sum }}{\left(\bar{y_{\cdot J}}-\bar{y_{..}}\right)}^{2}$$

(16) $$SSAB=\overset{a}{\underset{i=1}{\sum }}\overset{b}{\underset{j=1}{\sum }}n{\left(\bar{y_{lJ}}-\bar{y_{..}}\right)}^{2}-SSA-SSB$$

(17) $$SSR=\overset{a}{\underset{i=1}{\sum }}\overset{b}{\underset{j=1}{\sum }}{\left(y_{ijk}-\bar{y_{lJ}}\right)}^{2}$$

**Table 3:** Key elements of two-way ANOVA calculations.

Source of Variation	Sum of Squares	Degrees of Freedom	Mean Squares	F-Value	p-Value
Factor A	SSA (attributable to factor A)	a−1	$\frac{SSA}{a-1}$	$F_{A\left(a-1,ab\left(n-1\right)\right)}=\frac{\frac{SSA}{a-1}}{\frac{SSE}{ab\left(n-1\right)}}$	$p_{A}$
Factor B	SSB (attributable to factor B)	b−1	$\frac{SSB}{b-1}$	$F_{B\left(b-1,ab\left(n-1\right)\right)}=\frac{\frac{SSB}{b-1}}{\frac{SSE}{ab\left(n-1\right)}}$	$p_{B}$
Interaction AB	SSAB (attributable to interaction AB)	$\left(a-1\right)\left(b-1\right)$	$\frac{SSAB}{\left(a-1\right)\left(b-1\right)}$	$F_{AB\left(\left(a-1\right)\left(b-1\right),ab\left(n-1\right)\right)}=\frac{\frac{SSAB}{\left(a-1\right)\left(b-1\right)}}{\frac{SSE}{ab\left(n-1\right)}}$	$p_{AB}$
Residues or error	SSE (attributable to residues)	$ab\left(n-1\right)$	$\frac{SSE}{ab\left(n-1\right)}$	F	p

### 2.3. K-Means Clustering Technique

K-means is an unsupervised clustering technique that separates all data into K clusters. Each data point is assigned to one of the K clusters that minimize the Euclidean distance between the data point and the cluster’s center. The centers are then updated, and the data points are reassigned to the closest center throughout several iterations. The iterations are repeated, until the centers do not move or the data points do not change the cluster to which they are assigned [55,56]. Algorithm 2 describes the K-means clustering technique.
**Algorithm 2.** K-means Algorithm1: Initialize the number of clusters 2: Choose initial cluster centers 3: **While** The stopping criterion is not satisfied, **do** 4:   Assign each data point to one cluster 5:   Update the center of each cluster 6: **End while**

## 3. Proposed Segmentation Method

**Figure 7:**
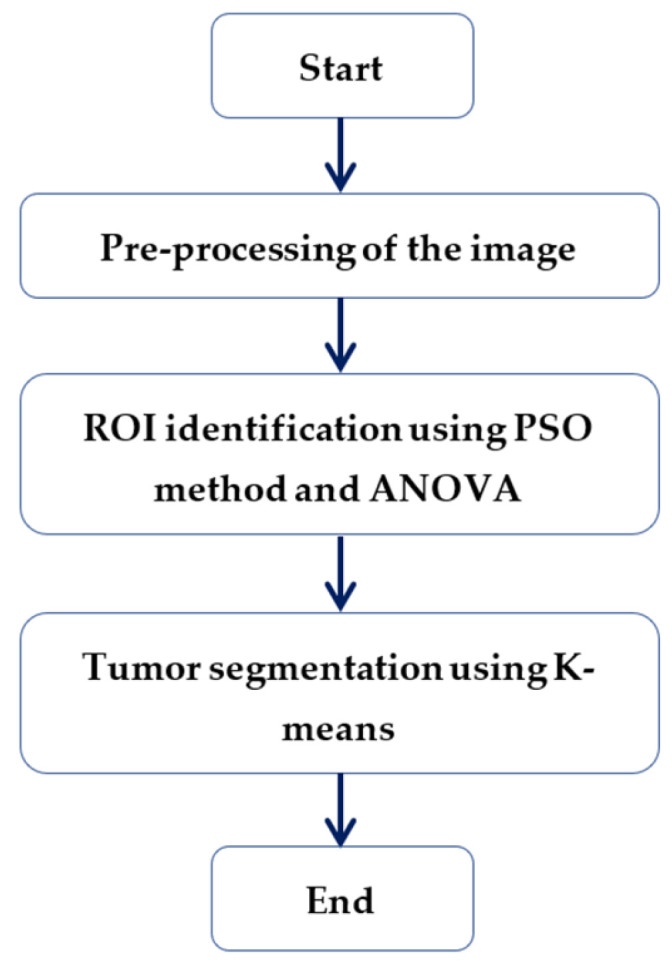
Diagram of the proposed approach.

**Figure 8:**
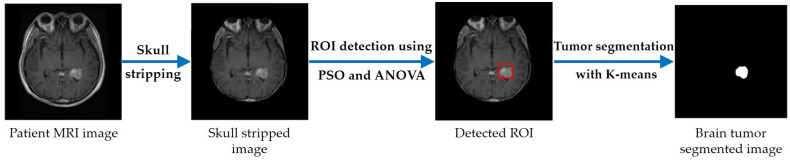
Flowchart of the proposed segmentation method.

The proposed segmentation method consists of three main stages. In the first stage, a pre-processing of the image is performed, to eliminate any unnecessary data. Then, PSO is applied, to identify the ROI, and K-means is used, to segment the ROI. An illustrative diagram is shown in Figure 7, and a simple flowchart of the proposed method is presented in Figure 8 where the detection and localization of the tumor is marked by the red square.

### 3.1. Image Pre-Processing

Image pre-processing is vital for removing noisy, inconsistent, incomplete, and irrelevant data [57]. Several approaches can improve the image quality during its transmission or storage [58]. We will mention, for example, those based on the concept of compressed sensing, where the image enhancement is carried out during acquisition [59,60,61,62,63]. An alternative approach is to perform simple and efficient de-noising (close to optimality) from first or second generation wavelets [64,65,66].

**Figure 9:**
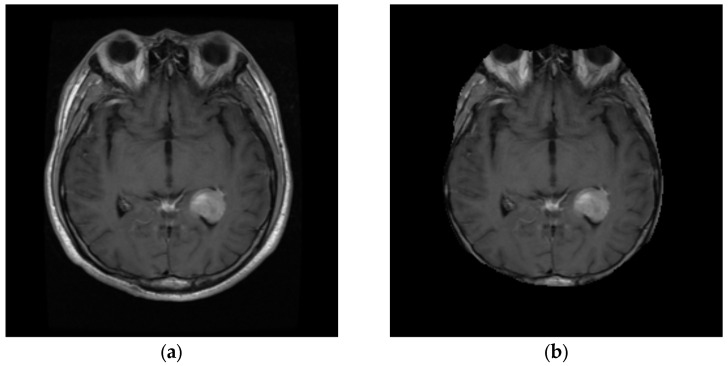
Pre-processing step: (**a**) original brain image; (**b**) brain image after pre-processing.

Skull-stripping is a crucial step in eliminating from the brain image all non-brain tissue, such as skull bone, fat, skin, etc. [67]. To this end, several approaches have been developed [68,69]. The first step of the proposed technique is a skull-stripping procedure, in which the gray-scale image is converted into a binary image, using a fixed threshold. Then, two morphological operations—filling and erosion—are applied to the binary image. Finally, the original image is masked by the obtained binary image; the generated image is the skull-stripped image (see Figure 9) [60,70,71,72].

### 3.2. ROI Detection

PSO is a powerful metaheuristic technique, designed to solve complex optimization problems. The proposed algorithm uses PSO to search for the optimal block containing the tumor within the MRI image. The principal concept of this stage is described as follows.

**Figure 10:**
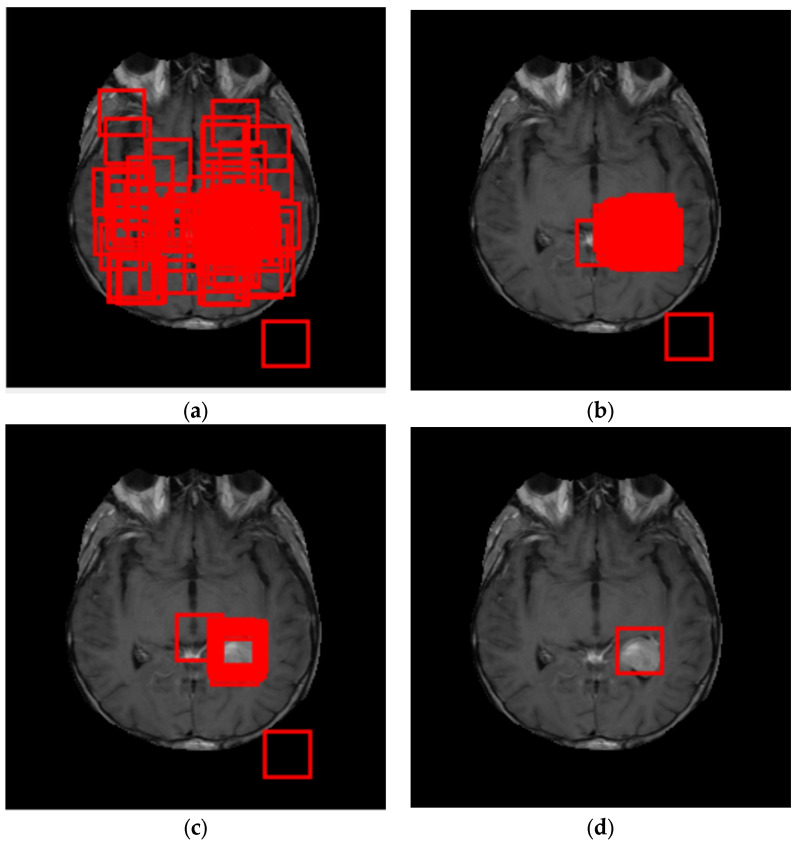
ROI identification, using PSO and ANOVA: (**a**) candidate blocks (in red) after 5 iterations; (**b**) candidate blocks after 15 iterations; (**c**) candidate blocks after 35 iterations; (**d**) candidate blocks after 50 iterations.

Firstly, several candidate blocks are randomly initialized within the MRI image, and each candidate block is evaluated with a fitness function, to determine the best blocks (see Figure 10). The proposed algorithm’s fitness function is based on the two-way ANOVA, to analyze variance. The used data are the candidate block and the MRI image of a no-disease brain. The used fitness function is given by (18).

(18) $$fitness=\frac{MSA}{MSE}+\frac{MSB}{MSE}$$

As MSA and MSB represent the variability among group means, and MSE represents the variability within the group, divided by the degree of freedom, then the high values of MSA and MSB correspond to significant variability between the candidate block and the no-disease brain image, which means that there is an abnormal tissue in this block. In other words, the large variability between the candidate block and the no-disease brain image signifies that this candidate block contains a tumor. Therefore, the candidate block that corresponds to the fitness function’s maximum value is considered the best block found. Once the fitness function evaluation and the global and individual best blocks have been updated, the positions of the candidate blocks are also updated, using (1) and (2). The fitness function evaluation and updating block processes are repeated, until maximum iterations are reached. As some solutions can be evaluated more than once in a metaheuristic technique, the fitness function value and the positions of each candidate solution are stored in a matrix, to reduce the computational time. Then, if the PSO algorithm attempts to evaluate an already-evaluated solution, its fitness value is taken directly from the matrix. This idea has decreased the computational complexity of block-matching problems [69].

### 3.3. Tumor Segmentation

**Figure 11:**
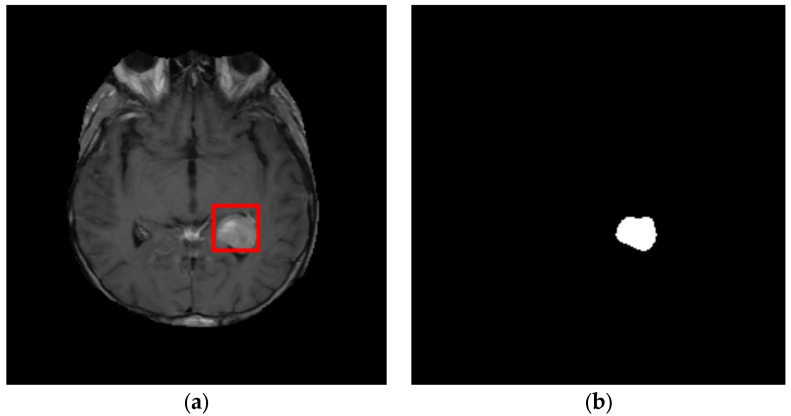
ROI segmentation using K-means: (**a**) identified ROI; (**b**) ROI segmented with K-means.

For tumor segmentation, the K-means clustering technique is applied to the global best block, found in the ROI detection stage, containing the tumor. Because we classify the blocks as tumor or non-tumor, the K-means method uses two clusters. Figure 11 illustrates the identified ROI and its segmentation using K-means.

**Figure 12:**
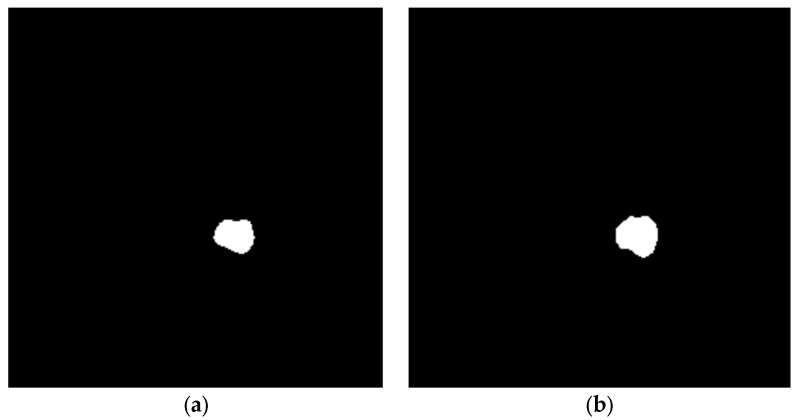
Performance comparison between the proposed brain tumor segmentation and Ground Truth: (**a**) segmentation using our method; (**b**) Ground Truth.

The segmentation results are then evaluated, by measuring the similarity or dissimilarity between them and the Ground Truth. Figure 12 illustrates the segmentation result of our approach and the Ground Truth.

Over the years, several similarity and dissimilarity metrics have been formulated and reported in the literature. To evaluate our algorithm, we used the following metrics [73]:

1. Dice similarity coefficient:
  (19) $$\frac{2TP}{2TP+FP+FN}$$

2. Jaccard distance:
  (20) $$\frac{TP}{TP+FP+FN}$$

3. Correlation coefficient:
  (21) $$\frac{1}{M\times N}\overset{M}{\underset{i=1}{\sum }}\overset{N}{\underset{j=1}{\sum }}\left(\frac{Is_{i,j}-Is}{\sigma_{Is}}\right)\left(\frac{Ig_{i,j}-Ig}{\sigma_{Ig}}\right)$$

4. Root Mean Squared Error (RMSE):

(22) $$\frac{1}{M\times N}\overset{M}{\underset{i=1}{\sum }}\overset{N}{\underset{j=1}{\sum }}{\left(Is_{i,j}-Ig_{i,j}\right)}^{2}$$

where FP was the number of false positives, TP was the number of true positives, FN was the number of false negatives, Is referred to the segmented image with our technique, Ig was the Ground Truth image, and M and N represented the image’s size.

As the Dice coefficient and Jaccard distance are two metrics of similarity, a powerful method should maximize these criteria. The correlation criterion is also a similarity metric, and varies between −1 and +1; the perfect positive correlation is achieved when the coefficient equals +1. RMSE is, on the other hand, a dissimilarity metric; the minimum value of RMSE is indicative of a robust segmentation technique [74].

## 4. Experimental Analysis

The proposed brain tumor segmentation approach was evaluated using the private CIKA [75] and the challenging BraTS 2015 [16] databases. This section presents the specifications of each employed database. Furthermore, we analyze the results obtained from our proposed approach, and compare the results with other classical approaches.

### 4.1. Experiments on the KICA Database

#### 4.1.1. Database Description

The proposed algorithm was implemented for the evaluation study on different MRI images and Ground Truths obtained from a database provided by the Kouba imaging center—Algiers (KICA) [75]—which included the brain tumor images and the corresponding Ground Truth images (complete tumor areas). In total, 223 people contributed to the constitution of the database (120 Train/103 Test). The disease-free brain images used were in Digital Imaging and Communications in Medicine (DICOM) format, and were selected in the same sections as the brain tumor images. We selected several MRI images, to perform our different experiments. The presented work was a collaboration between several institutions from Algeria and France. The Ground Truth MRI models were defined with the help of radiologists, neurologists, and biomedical engineers from the Kouba imaging center in Algiers (Algeria) and the Hospital of Tours (France).

#### 4.1.2. Experiments

This section is divided into two parts. The first part illustrates the essential role of ANOVA in our algorithm. The ANOVA-based fitness function results were compared to those obtained with the SAD fitness function. Following the same concept of variability explained above, the candidate block that gave the maximum SAD value was considered the tumor block. In the second part of this work, we compared the experimental results of our algorithm with several well-known segmentation techniques, such as fuzzy C-means (FCM), K-means, Otsu thresholding, local thresholding, and watershed segmentation.

A. Experiment #1

The robustness of any technique based on metaheuristics depends on the fitness function used: the latter is a decisive parameter in evaluating all the candidate solutions and determining the optimal global solution. In our segmentation technique, the fitness function measured the variability or the difference between the candidate blocks in the brain tumor image and the corresponding blocks in the no-disease image; a significant difference between them indicated the existence of a tumor. We resorted to the statistical method ANOVA, using the fitness function expressed in (18). As the difference could also be measured with any dissimilarity criterion, we changed the used fitness function of (18), and replaced it with the SAD criterion, in order to prove the efficiency of our ANOVA-based fitness function. The SAD metric, also called the L1 norm or Manhattan norm, is a dissimilarity criterion, used to compare the intensities of two blocks or images [74], and is defined as follows:

(23) $$SAD=\overset{M}{\underset{i=1}{\sum }}\overset{N}{\underset{j=1}{\sum }}\left|I_{1_{i,j}}-I_{2_{i,j}}\right|$$

where $I_{1}$ and $I_{2}$ represent the candidate blocks in the brain tumor image and the no-disease image.

**Figure 13:**
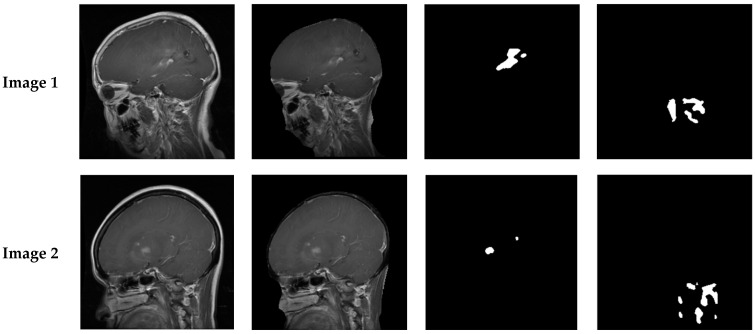

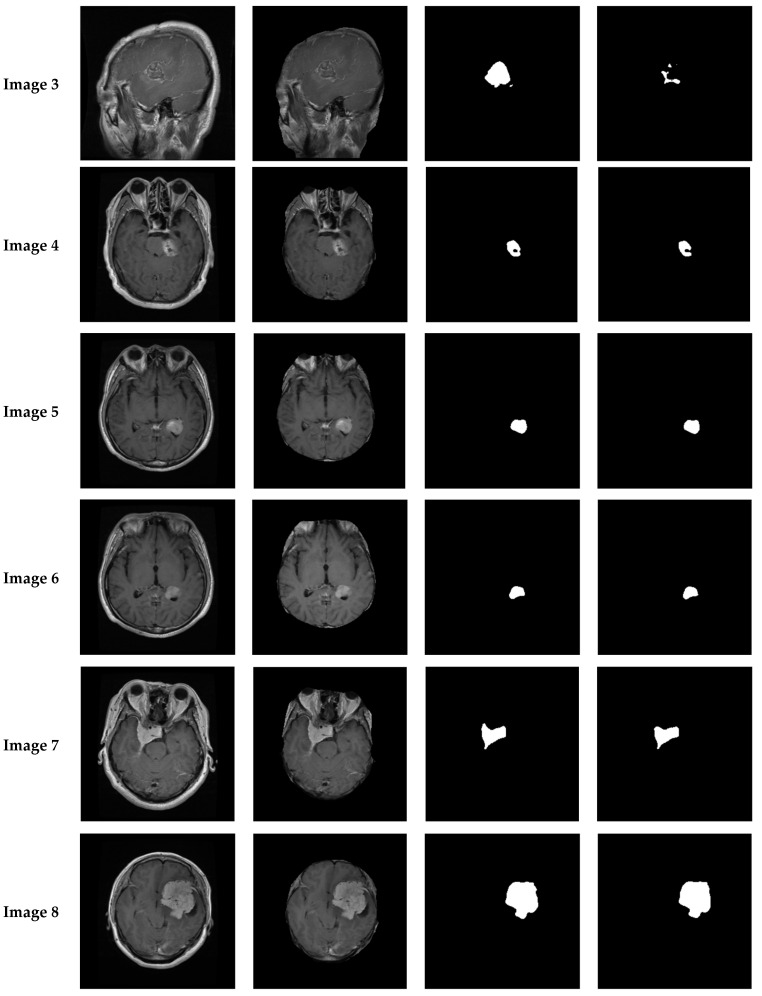

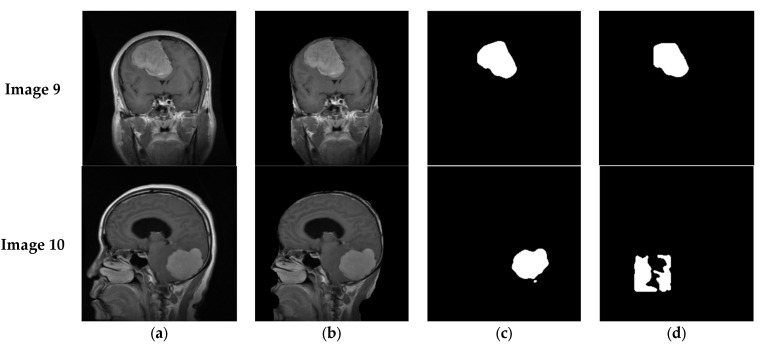
The efficiency of brain tumor segmentation on the KICA dataset: comparison of ANOVA and SAD-based methods. (**a**) Original images. (**b**) Pre-processing. (**c**) Our method with ANOVA. (**d**) Our method with SAD.

A high SAD value showed a substantial difference between the candidate block and the no-disease image, indicating the presence of a tumor. Therefore, the candidate block that gave the maximum SAD value was considered the tumor block. Figure 13 shows the original images before and after the pre-processing and segmentation procedures, where the used fitness function was based on ANOVA and the SAD criterion.

Table 4 shows the Dice, Jaccard distance, correlation, and the RMSE values obtained with the two fitness functions tested by our segmentation method. From Figure 13, we can observe that using SAD as a fitness function gave non-relevant segmentation results in several cases; however, the segmentation results with ANOVA were near the Ground Truths with most of the tested images. In addition, we can observe from Table 4 that the results of segmentation with ANOVA were higher, and near to 100% in most of the tested cases using the Dice similarity coefficient, Jaccard distance, and correlation coefficient, and near to 0% with the RMSE metric. In contrast, SAD gave some promising results, but the majority were not satisfactory. As a recapitulation, it is clear that using ANOVA as a fitness function yielded much better results than those obtained with the SAD fitness function, demonstrating the robustness and effectiveness of the technique in evaluating candidate blocks.

B. Experiment #2

**Figure 14:**
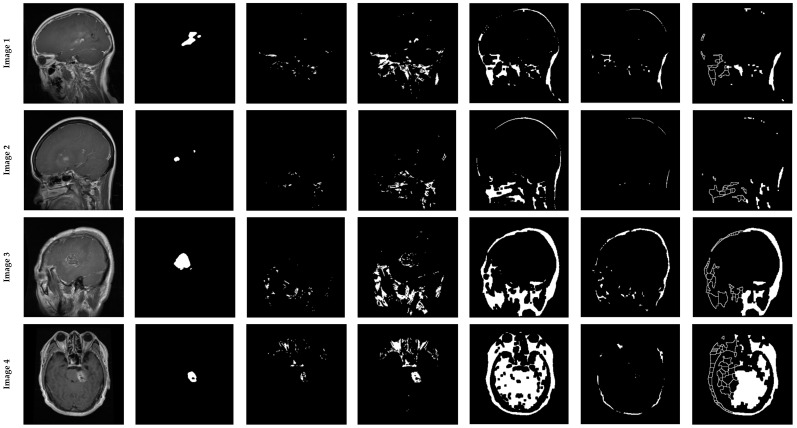

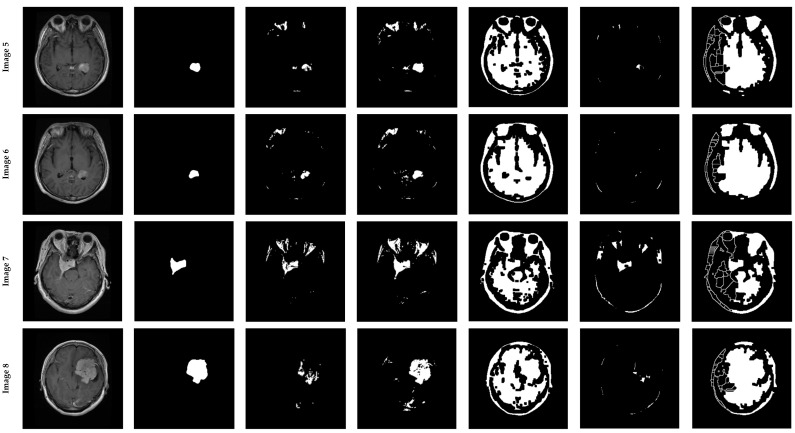

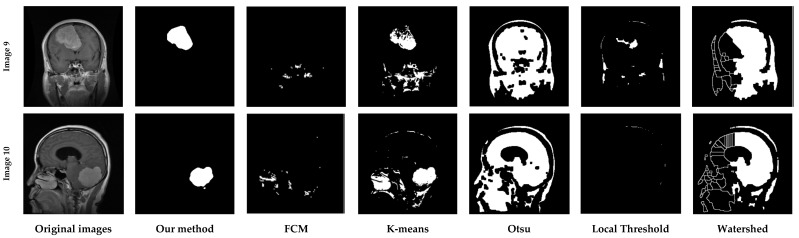
The efficiency of brain tumor segmentation on the KICA dataset: comparison between the proposed ANOVA-based method and other well-known methods.

We assessed our algorithm’s effectiveness against several well-known segmentation techniques in the second experiment. The segmented images obtained with our method, and other segmentation methods, are shown in Figure 14. In addition, Table 5, Table 6, Table 7 and Table 8 highlight the statistical results and comparisons of our method and other related methods, using the following metrics: Dice similarity coefficient; Jaccard distance; correlation coefficient; and RMSE metric.

It can be observed, from the qualitative and quantitative comparisons, that the proposed method can segment the brain tumor very efficiently, in contrast to the classical methods, where their segmentations are not relevant or not satisfactory in the majority of cases. The power of our method resides primarily in the following point: the first stage of our algorithm, that determines the ROI, then segments only this region, making the brain tumor segmentation results free of any irrelevant information, such as skull bone. Unlike other segmentation techniques, such as those based on Otsu or local thresholding, some extraneous information remains on the segmented image.

**Figure 15:**
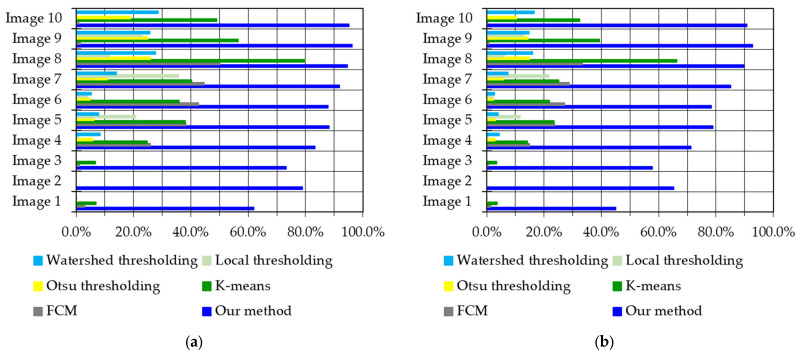

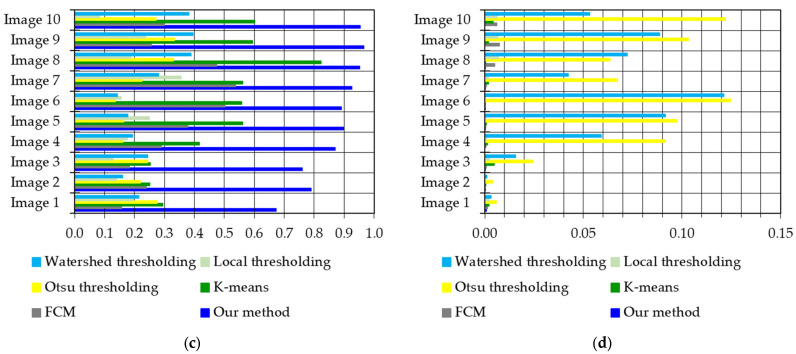
Comparison of segmentation results on the CIKA dataset based on: (**a**) Dice similarity coefficient; (**b**) Jaccard Distance; (**c**) correlation coefficient; (**d**) RMSE metric.

To complement the above results, Figure 15 further highlights the robustness and effectiveness of our proposed method. As shown in Figure 15a–d, the results of our algorithm (blue bars in each figure) are highest in Dice, Jaccard, and correlation, and lowest in RMSE, compared to other competing techniques, which proves the efficiency and robustness of our method. Thus, all the experimental results demonstrate that the proposed method outperforms all other competing methods.

### 4.2. Experiments on the BraTS 2015 Database

To ensure that our previous findings could be generalized, we used an external and very challenging dataset to test the performance of the PSO–ANOVA-based segmentation approach.

#### 4.2.1. Database Description

The Medical Image Computing and Computer Assisted Intervention (MICCAI) conference provided the BraTS database [16]; it is the official database for the conference’s brain tumor MRI segmentation challenge, and it is also commonly used by researchers working on brain tumor MRI segmentation. The BraTS database has been updated annually since the challenge was launched in 2012.

**Figure 16:**
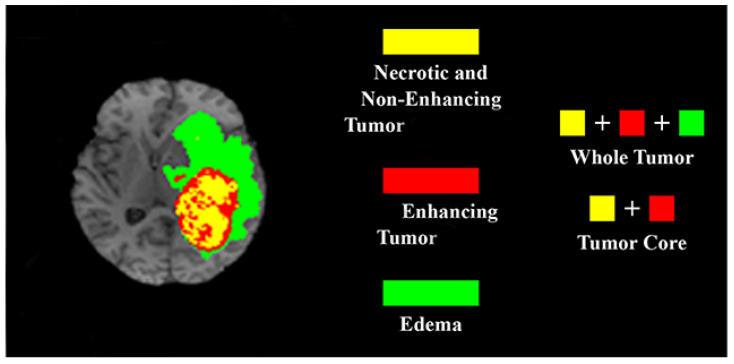
The three modalities of tumor regions: complete, core, and enhancing tumors.

The dataset for segmenting brain tumor images was called BraTS 2015. It consisted of 54 low-grade gliomas (LGG) and 220 high-grade gliomas (HGG) MRIs. These 274 images were reserved for the training set, while 110 were for the testing set. The total size of all the MRI images was 240 × 240 × 155. The four MRI modalities were T1, T1c, T2, and T2-FLAIR. Four intra-tumoral classes—edema, enhancing tumor, non-enhancing tumor, and necrosis—were provided as segmented ‘Ground Truth’. The Dice similarity coefficient was used in this experiment to evaluate and compare the segmentation results with some recently published state-of-the-art methods. The three modalities of tumor regions—complete, core, and enhancing tumors—were considered, in computing the performance measure. The complete (or whole) tumor area comprised enhancing and non-enhancing cores, edema, and necroses. Necroses and enhancing and non-enhancing cores were all in the core region. Only the area of the enhancing region was referred to as the enhancing tumor. Figure 16 highlights the three modalities of tumor regions. The Dice score was determined by superimposing the anticipated output image over the manually segmented label (i.e., Ground Truth).

#### 4.2.2. Experiments

**Figure 17:**
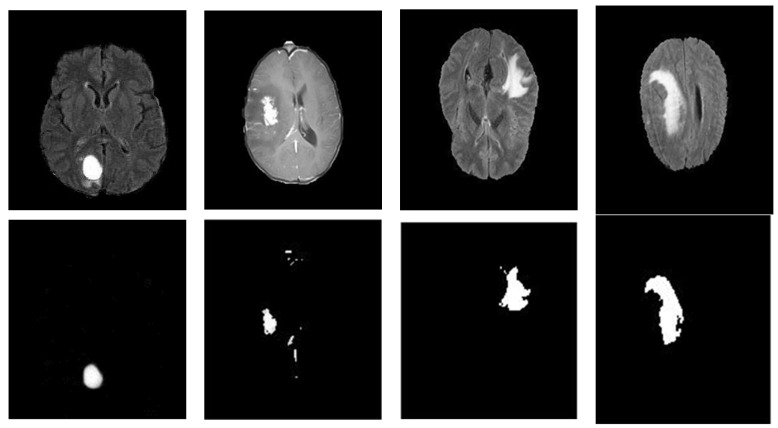
The efficiency of our proposed brain tumor segmentation method on the BraTS 2015 dataset: (top row) original T2-FLAIR images; (bottom row) segmentation results of complete tumors.

As we did with the KICA dataset, we conducted visual and quantitative experiments on the BraTS 2015 dataset, to assess the performance of our proposed brain tumor segmentation approach. A subset of four T2-FLAIR images, representing the most clinically encountered tumors, was chosen, to highlight the visual assessment; Figure 17 shows the results of our proposed brain tumor segmentation approach, using HGG and LGG MRI images. According to the WHO, grades I and II are considered low-grade glioma (LGG), while grades III and IV are highly malignant, and are called high-grade glioma (HGG). We note that the type of segmentation considered in this demonstration is based on complete tumors, which include necrosis, edema, and enhancing and non-enhancing cores. Furthermore, Table 9 presents our approach’s performance, using the Dice similarity coefficient as a statistical criterion, and compares our results against the results of recently published approaches related to the BraTS 2015 challenging dataset. To make a comparison against the state-of-the-art, we have reported in Table 9 the segmentation results of the three modalities of tumor regions (i.e., complete, core, and enhancing tumors).

From the visual and statistical evaluations, we can observe that our approach gave relevant segmentation results in most tested cases, and that the segmentation results were near the Ground Truth images. Besides, we can observe from Table 9 that the segmentation results were higher (mean ≈87%) with the three modalities of tumor regions using the Dice similarity coefficient. According to the complete regions, our approach outperformed all compared state-of-the-art approaches, like CNN [76,77,78] and ILinear [39], while producing equivalent and competitive outcomes to the core and enhancing tumor areas. Finally, as the findings obtained were similar to the results obtained with the CIKA dataset, we can deduce that our approach is a robust and effective technique.

As the majority of the PSO–ANOVA results were highly compatible with private and public datasets, we can deduce that the performance of our brain tumor segmentation approach is satisfactory, and we are confident that it can be implemented in real-world applications, to help doctors in making clinical decisions.

## 5. Conclusions

In this paper, a new method for brain tumor segmentation has been proposed. This method consists of four steps:

- The skull bone is precisely removed from the image, to exclude irrelevant data.
- The particle swarm optimization (PSO) technique is then applied, to detect the region of interest (ROI) that contains the brain lesion.
- The fitness function used to evaluate the candidate blocks is based on a two-way fixed-effects analysis of variance (ANOVA).
- Finally, in the last step of the method, the K-means segmentation method is used in the lesion block, to classify it into two possible categories: tumor and non-tumor.

An evaluation study was performed using extensive magnetic resonance imaging (MRI) databases; a visual assessment and four statistical measures were used to evaluate the performance of the tumor segmentation. The images representing the most clinically encountered positions were used for the visual assessment. The results show that competing approaches do not provide usable segmentation results in some cases, whereas our approach is a promising solution for clinical decision support. Indeed, statistically, the results show that the proposed method gives a tumor segmentation accuracy of 96%, outperforming other state-of-the-art methods. Moreover, comparing our method with the manual and careful segmentation performed by experts to obtain what is called ‘Ground Truth’ does not show significant differences. Indeed, the difference is about 1%.

The different results, and the comparison with state-of-the-art methods, show that our approach can be a useful tool for brain cancer detection, diagnosis, and radiotherapy treatment planning. The future direction of our research in brain tumor segmentation must address the limitations of the unsupervised approach by: (1) combining PSO, ANOVA, and a CNN model [84,85,86,87,88,89,90]; (2) using generative adversarial networks [91,92,93,94] to pre-process, colorize, correct, and enhance images before presenting them to the segmentation algorithm.

## Figures and Tables

**Table 1:** Analysis of basic MRI sequences in the context of brain tumors.

Tissue	T1-Weighted	T2-Weighted	FLAIR
White Matter	Light	Dark Gray	Dark Gray
Fat	Bright	Light	Light
CSF	Dark	Bright	Dark
Inflammation	Dark	Bright	Bright
Cortex	Gray	Light Gray	Light Gray

**Table 4:** The results of our segmentation method on the KICA dataset, using the Dice similarity coefficient, Jaccard distance, correlation coefficient, and RMSE metric with two fitness functions: ANOVA and SAD.

Images	Dice Similarity Coefficient (%)		Jaccard Distance (%)		Correlation Coefficient (−1 to +1)		RMSE Metric (%)	
	ANOVA	SAD	ANOVA	SAD	ANOVA	SAD	ANOVA	SAD
Image 1	62.008%	NRS	44.936%	NRS	0.673064	0.475478	0.0006647	0.0010605
Image 2	78.997%	NRS	65.285%	NRS	0.790116	0.386167	0.0000158	0.0014934
Image 3	73.276%	24.432%	57.823%	13.916%	0.761069	0.532032	0.0003579	0.0005049
Image 4	83.265%	78.242%	71.329%	64.26%	0.870553	0.820469	0.0000657	0.0001091
Image 5	88.252%	87.581%	78.974%	77.907%	0.897922	0.888510	0.0000398	0.0000464
Image 6	87.844%	85.748%	78.323%	75.051%	0.891440	0.865518	0.0000243	0.0000361
Image 7	91.919%	88.809%	85.046%	79.871%	0.925806	0.903027	0.0000686	0.0001325
Image 8	94.535%	93.593%	89.637%	87.959%	0.952524	0.942536	0.0001223	0.0002402
Image 9	96.211%	90.469%	92.699%	82.598%	0.953663	0.66343	0.0000498	0.0004337
Image 10	95.154%	NRS	90.756%	NRS	0.950669	0.94286	0.0000414	0.0133088

NRS signifies « non-relevant segmentation ».

**Table 5:** Comparison between the results of our segmentation method on the KICA dataset and some well-known segmentation techniques using the Dice similarity coefficient.

Images	Our Method	FCM	K-Means	Otsu Thresholding	Local Thresholding	Watershed Thresholding
Image 1	62.008%	2.9106%	6.858%	0.044%	0.303%	NRS
Image 2	78.997%	NRS	NRS	NRS	NRS	NRS
Image 3	73.276%	1.127%	6.690%	NRS	NRS	NRS
Image 4	83.265%	25.948%	24.823%	5.750%	0.906%	8.297%
Image 5	88.252%	38.213%	38.137%	6.130%	20.762%	7.738%
Image 6	87.844%	42.716%	35.910%	4.683%	4.088%	5.357%
Image 7	91.919%	44.635%	40.291%	10.921%	35.751%	13.968%
Image 8	94.535%	50.052%	79.763%	25.940%	11.712%	27.638%
Image 9	96.211%	1.476%	56.53%	25.038%	23.014%	25.643%
Image 10	95.154%	0.810%	48.957%	18.878%	NRS	28.530%

NRS signifies « non-relevant segmentation ».

**Table 6:** Comparison between the results of our segmentation method on the KICA dataset and some well-known segmentation techniques using the Jaccard distance.

Images	Our Method	FCM	K-Means	Otsu Thresholding	Local Thresholding	Watershed Thresholding
Image 1	44.936%	1.4768%	3.551%	0.022%	0.151%	NRS
Image 2	65.285%	NRS	NRS	NRS	NRS	NRS
Image 3	57.823%	0.566%	3.461%	NRS	NRS	NRS
Image 4	71.329%	14.908%	14.17%	2.96%	0.455%	4.328%
Image 5	78.974%	23.619%	23.561%	3.161%	11.583%	4.025%
Image 6	78.323%	27.159%	21.885%	2.398%	2.087%	2.752%
Image 7	85.046%	28.729%	25.228%	5.776%	21.766%	7.508%
Image 8	89.637%	33.38%	66.339%	14.903%	6.220%	16.035%
Image 9	92.699%	0.7438%	39.402%	14.310%	13.003%	14.707%
Image 10	90.756%	0.406%	32.413%	10.423%	NRS	16.639%

NRS signifies « non-relevant segmentation ».

**Table 7:** Comparison between the results of our segmentation method on the KICA dataset and some well-known segmentation techniques using the correlation coefficient.

Images	Our Method	FCM	K-Means	Otsu Thresholding	Local Thresholding	Watershed Thresholding
Image 1	0.673064	0.157563	0.293867	0.275414	0.196262	0.214095
Image 2	0.790116	0.239039	0.250315	0.219895	0.138782	0.160484
Image 3	0.761069	0.182389	0.253495	0.243609	0.129738	0.243366
Image 4	0.870553	0.289513	0.417104	0.159479	0.175391	0.192943
Image 5	0.897922	0.378542	0.561895	0.164128	0.249723	0.177235
Image 6	0.891440	0.502674	0.557390	0.135842	0.154660	0.142385
Image 7	0.925806	0.537026	0.560681	0.224682	0.356487	0.281344
Image 8	0.952524	0.474860	0.822597	0.328776	0.188492	0.3892
Image 9	0.966158	0.255578	0.593773	0.333411	0.236488	0.3951
Image 10	0.953663	0.300080	0.602117	0.271452	0.084133	0.3824

**Table 8:** Comparison between the results of our segmentation method on the KICA dataset and some well-known segmentation techniques using the RMSE metric.

Images	Our Method	FCM	K-Means	Otsu Thresholding	Local Thresholding	Watershed Thresholding
Image 1	0.0006647	0.0012972	0.0021345	0.0057836	0.0020789	0.0031144
Image 2	0.0000158	0.0000920	0.0005815	0.0040139	0.0001327	0.0011636
Image 3	0.0003579	0.0008458	0.0046264	0.0242370	0.0024103	0.0155236
Image 4	0.0000657	0.0005798	0.0013554	0.0915980	0.0010545	0.0589136
Image 5	0.0000399	0.0005223	0.0006572	0.0973590	0.0006525	0.0915326
Image 6	0.0000243	0.0002296	0.0003564	0.1246628	0.0005443	0.1213210
Image 7	0.0000686	0.0007301	0.0018533	0.0672948	0.0017184	0.0423277
Image 8	0.0001223	0.0049038	0.0004566	0.0636407	0.0068672	0.0722
Image 9	0.0000498	0.0073548	0.0018346	0.1033327	0.0065827	0.0887
Image 10	0.0000414	0.0060980	0.0040885	0.1220065	0.0062485	0.0533

**Table 9:** The results of our segmentation method on the BraTS 2015 dataset, using the Dice similarity coefficient applied on complete, core, and enhancing tumors, and comparison to the state-of-the-art.

Authors	Year	Methods	Dice		
			Complete	Core	Enhancing
Havaei et al. [76]	2016	CNN (Two-Phase Patch-Wise Training Procedure)	88%	79%	73%
Pereira et al. [77]	2016	CNN	87%	73%	68%
Tseng et al. [78]	2017	CNN (Encoder-Decoder Architecture)	85%	68%	68%
Hussain et al. [39]	2018	ILinear	86%	87%	90%
Iqbal et al. [79]	2018	CNN (Sequential Multiple Neural Network Layers)	87%	86%	79%
Liu et al. [80]	2018	CNN (ResNet-50)	87%	62%	68%
Hu and Deng [81]	2019	MCCNN + CRFs	87%	76%	75%
Li et al. [82]	2019	CNN (Modified U-Net Architecture)	89%	73%	73%
Elmezain et al. [83]	2022	CapsNet + LDCRF + Post-processing	91%	86%	85%
Atia et al.	2022	Our Method	91%	87%	86%

## Data Availability

The data can be shared up on request.

## Abbreviations

The following abbreviations and acronyms are used in this manuscript:

ANOVA	Analysis of Variance
BRATS	Multimodal Brain Tumor Segmentation Challenge
CNN	Convolutional Neural Networks
CSF	Cerebrospinal Fluid
DICOM	Digital Imaging and Communications in Medicine
FCM	Fuzzy C-Means
FLAIR	Fluid Attenuated Inversion Recovery
FN	False Negatives
FP	False Positives
GA	Genetic Algorithm
Gd	Gadolinium-based Contrast Agents
HGG	High-Grade Gliomas (HGG)
LGG	Low-Grade Gliomas (LGG)
KICA	Kouba Imaging Center—Algiers
MRI	Magnetic Resonance Imaging
RMSE	Root Mean Squared Error
PSO	Particle Swarm Optimization
ROI	Region of Interest
SVM	Support Vector Machines
SA	Simulated Annealing
SAD	Sum-of-Absolute-Differences
SSE	Error Sum of Squares
SSF	Factorial Sum of Squares
SSR	Residual Sum of Squares
SST	Total Sum of Squares
T1	T1-Weighted Imaging Sequence
T2	T2-Weighted Imaging Sequences
T1c	T1-Weighted Contrast-Enhanced
TE	Echo Time
TR	Repetition Time
TP	True Positives
WHO	World Health Organization
